# The prognostic value of CXCR4 PET/CT imaging in unilateral primary aldosteronism patients after adrenalectomy

**DOI:** 10.1186/s13550-025-01242-6

**Published:** 2025-04-17

**Authors:** Shuai Shao, Haozhe Xu, Zhuo Xing, Yulong Hong, Xuan Yin, Jianguang Luo, Kai Ai, Xin Su, Xiaowei Ma, Yuan Li

**Affiliations:** 1 Department of Urology, The Second Xiangya Hospital, Central South University, Changsha, Hunan 410011 China; 2 Department of Nuclear Medicine, The Second Xiangya Hospital, Central South University, Changsha, Hunan 410011 China; 3 Department of Radiology, The Second Xiangya Hospital, Central South University, Changsha, Hunan China; 4 Department of Endocrinology, The Second Xiangya Hospital, Central South University, Changsha, Hunan 410011 China

**Keywords:** Primary aldosteronism, CXCR4 PET/CT, Adrenalectomy, Clinical outcome

## Abstract

**Background:**

CXCR4 PET/CT imaging has emerged as a tool for diagnosis and subtyping of primary aldosteronism (PA). But its prognostic value for postoperative blood pressure recovery has not been fully discussed.

**Results:**

The lesional SUVmax to the contralateral adrenal tissue SUVmean ratio (LCR) was identified as an independent predictor of clinical success at both the 3-month and 6-month assessments. The AUC for LCR was 0.894 at the 3-month and 0.832 at the 6-month. Patients were divided into high and low LCR groups according to the optimal cut-off of 3.240. The high LCR group exhibited elevated CXCR4 and CYP11B2 expression, higher PAC level, a greater probability of achieving complete clinical success compared to the low LCR group. Moreover, LCR was correlated with lateralization index and contralateral suppression index.

**Conclusions:**

LCR is a reliable independent predictor of postoperative blood pressure recovery in PA. Patients with LCR over 3.240 may benefit more from adrenalectomy. We recommend increased utilization of CXCR4 PET/CT for patients with PA.

**Registration:**

ChiCTR2200062844. Registered 20 August 2022.

**Supplementary Information:**

The online version contains supplementary material available at 10.1186/s13550-025-01242-6.

## Background

Primary aldosteronism (PA), the most prevalent cause of endocrine hypertension, is characterized by dysregulated aldosterone production independent of renin, volume, or sodium loading [1, 2]. PA patients encounter a higher incidence of cardiovascular events compared to patients with essential hypertension of the same blood pressure grade, encompassing stroke, non-fatal myocardial infarction, and atrial fibrillation [3, 4]. For patients with unilateral primary aldosteronism (UPA), adrenalectomy is the recommended treatment, while bilateral PA patients are usually managed with mineralocorticoid receptor antagonists [5, 6].

Currently, the primary diagnostic and subtyping methods for PA involve computed tomography (CT) and adrenal vein sampling (AVS) [7]. However, CT is hindered by low accuracy and cannot show functional status, while AVS faces limitations due to invasiveness and operational challenges [5]. Recently, positron emission tomography/computed tomography (PET/CT) has emerged as a non-invasive method for PA diagnosis and subtyping. In a prospective cohort, a PET tracer ^11^C-metomidate targeting the aldosterone synthase CYP11B2 demonstrated diagnostic efficacy comparable to AVS [8]. Nevertheless, the application of ^11^C-metomidate is constrained by low molecular specific selectivity for CYP11B2 and CYP11B1, so patients need dexamethasone preparation before imaging to inhibit the combination of trace and CYP11B1. Moreover, the production of ^11^C-metomidate is difficult owing to the short half-life of ^11^C [9].

The PET tracer ^68^Ga-Pentixafor targets CXC chemokine receptor type 4 (CXCR4), a transmembrane G protein-coupled receptor with significantly higher expression in aldosterone-producing adenomas (APA) than in normal adrenal tissue and nonfunctional adenomas. The expression level of CXCR4 strongly correlates with the aldosterone synthase CYP11B2 in APA, so ^68^Ga-Pentixafor is expected to serve as a stable PET tracer without special preparation [10]. The reliable diagnostic and subtyping capacity of CXCR4 PET/CT in PA patients was demonstrated in previous studies [11, 12]. Furthermore, the potential relationship between PET values and the clinical outcome of PA was suggested [12–14]. However, it is important to note that these studies only implied an association between high PET values and favorable clinical outcome; no confirmation regarding the predictive value and accuracy of PET values for clinical outcome was provided. Consequently, the clinical applicability of these findings remains uncertain. Particularly, the question of whether CXCR4 PET/CT serves as an independent predictor for clinical outcome remains unexplored. For better clinical management, further exploration for the outcome prediction value of CXCR4 PET/CT in PA patients is necessary. Moreover, establishing a cut-off point based on CXCR4 PET/CT is crucial for accurately identifying patients who are likely to respond favorably to adrenalectomy.

In our previous study, we identified an association between PET values and postoperative outcomes of PA. However, our results were not statistically significant due to limited sample size and inappropriate outcome settings [15]. In this prospective study, we have increased the number of enrolled patients and refined the study design to determine whether the uptake values of CXCR4 PET/CT can serve as effective predictors of postoperative blood pressure recovery.

## Methods

### Study design and participants

Ethics approval was obtained from ethics committees at the Second Xiangya Hospital, and written informed consent was acquired from all participants. This prospective study was conducted at the Second Xiangya Hospital. Patients with PA clinical diagnosis determined by an experienced endocrinologist and willing to go through CXCR4 PET/CT were included from September 1, 2022 to September 1, 2023. Patients were eligible if they confirmed PA (according to the Endocrine Society guidelines) and were enable for adrenalectomy [5]. The selection of surgical treatment is primarily based on the discussions of the multidisciplinary diagnosis and treatment team, guided by the patient’s examination results. The main reference indicators are the findings from 68Ga-Pentixafor PET/CT. Exclusion criteria included suspicion of bilateral primary aldosteronism or adrenocortical carcinoma, familial PA due to germline mutations, and contraindications to CXCR4 PET/CT such as pregnancy or claustrophobia.

The clinical features including age, gender, BMI, duration of hypertension, duration of hypokalemia, adrenal CT imaging, serum potassium, aldosterone-to-renin ratio (ARR), plasma aldosterone concentration (PAC), systolic pressure and diastolic pressure (ARR and PAC were measured in sitting position) were collected.

### CXCR4 PET/CT imaging and analysis

^68^Ga-Pentixafor was synthesized in a sterile environment following the reported labeling approach [10]. PET/CT imaging with ^68^Ga-Pentixafor was conducted using a Siemens Biograph mCT PET/CT scanner (Siemens Medical Solutions, Erlangen, Germany). No special diet or preparation is required before PET/CT imaging. Static images from the head to mid-thigh were collected for 10 min, after 25 min of intravenous administration of ^68^Ga-Pentixafor (mean 88 ± 15 MBq; range, 74–111 MBq). A corresponding attenuation-corrected CT scan of the adrenal gland was conducted using a low-dose regimen. Fusion PET and low-dose CT images were acquired to assess the uptake of ^68^Ga-Pentixafor. None of the patients reported significant discomfort.

Two experienced nuclear medicine physicians independently analyzed all PET scans without access to clinical data. Discrepancies were resolved through consensus between the two radiologists. Based on visual analysis, a PET/CT lesion was considered positive when the adrenal nodule(s) exhibited higher uptake than the contralateral and adjacent normal adrenal glands. Quantitative analyses were performed using PMOD 4.3 software (PMOD TECHNOLOGIES, Zurich, Germany). The calculations included determining the maximum standardized uptake values (SUVmax) of the adrenal lesions, the lesional SUVmax to the normal liver SUVmean ratio (LLR), and the lesional SUVmax to the contralateral adrenal tissue SUVmean ratio (LCR).

### Adrenal vein sampling

Medications that may influence renin or aldosterone level were withdrawn at least 2 weeks before AVS. AVS was performed after 3 h in recumbent position. Adrenocorticotropin (ACTH) was injected as a 0.125 mg (25 U) bolus. Blood samples were obtained from inferior vena cava (IVC) and both adrenal veins (AVs) before and 10 min after ACTH stimulation. Successful catheterization was defined if selectivity index (SI, ratio of AV cortisol to IVC cortisol) ≥ 2 before and ≥ 3 after ACTH stimulation. Lateralization index (LI) was defined as the ratio of the dominant adrenal aldosterone/cortisol to the non-dominant adrenal aldosterone/cortisol. UPA was defined if post ACTH stimulation LI ≥ 4. The post ACTH stimulation LI was used for final analysis. Contralateral suppression index (CSI) was defined as the ratio of the non-dominant adrenal aldosterone/cortisol to the IVC aldosterone/cortisol before ACTH stimulation. Contralateral suppression was considered if CSI < 1.

### Patients management and follow-up

All included patients underwent CXCR4 PET/CT. The patient management was selected between adrenalectomy and medical therapy by a multidisciplinary team of endocrinologists, radiologists, and urologists. Patients who underwent adrenalectomy with at least 6 months post-surgical follow-up were included for analysis.

ARR, PAC, systolic pressure, diastolic pressure, and antihypertensive drug use were followed up at 3 and 6 months after adrenalectomy. ARR and PAC were measured in the sitting position. Blood pressure was measured according to the European Society of Hypertension/European Society of Cardiology guidelines for the management of arterial hypertension [16]. Clinical and biochemical outcomes were assessed following the PASO criteria at both 3 and 6 months after adrenalectomy [17]. Moreover, blood pressure recovery, including blood pressure and antihypertensive medication, was followed up weekly.

### Immunohistochemistry

Immunohistochemical analyses were conducted using paraffin-embedded specimens from subjects who underwent unilateral adrenal excision. The primary antibodies used were CXCR4 and CYP11B2. Adrenal sections were subjected to immunohistochemical staining using an automatic immunostaining apparatus. In the semi-quantitative analysis, staining intensity was scored as 0 (negative), 1 (weak), 2 (moderate), and 3 (strong). The percentage of positive cells was scored as follows: 0% for score 0, 1-9% for score 1, 10-49% for score 2, 50-74% for score 3, and 75-100% for score 4. The semi-quantitative h score was calculated by multiplying the intensity score with the percentage score. The h score was categorized into three levels: low (0–4), medium [4–8], and high [8–12]. The diagnosis of primary aldosteronism (PA) was based on the histology of primary aldosteronism (HISTALDO) consensus [18, 19].

### Statistical analysis

The patient features were summarized as frequencies (percentages) for categorical variables, and medians (interquartile ranges, IQRs) for continuous not normally distributed variables. The Mann-Whitney test was used for not normally distributed variables, while the Fisher exact test and the chi-square test were used for categorical variables.

Univariate and multivariate logistic regression analyses were employed to assess the association between ^68^Ga-Pentixafor uptake and clinical success. Multivariable logistic regression was performed for variables with *p* < 0.05 on univariable logistic regression and summarized as odds ratios (ORs, OR > 1 suggests that the factor serves as a protective element for clinical success) and 95% confidence intervals (CIs).

A Receiver Operating Characteristic (ROC) curve was utilized to measure the accuracy of LCR in predicting clinical success and determine the optimal cut-off value. Patients were then categorized into high and low LCR groups based on this cut-off. 1000 bootstrap samples were used for internal validation with ‘fbroc’ r package. The Kaplan–Meier method was employed to assess the disparity in the probability of attaining complete clinical success between the high and low LCR groups. Additionally, the Spearman correlation was utilized to examine the association between not normally distributed variables.

Statistical analyses were performed using R version 4.3.2. and GraphPad Prism 8.3. All statistical tests were two-sided with significance set at *p* < 0.05.

## Results

### Baseline and features of patients

**Fig. 1:**
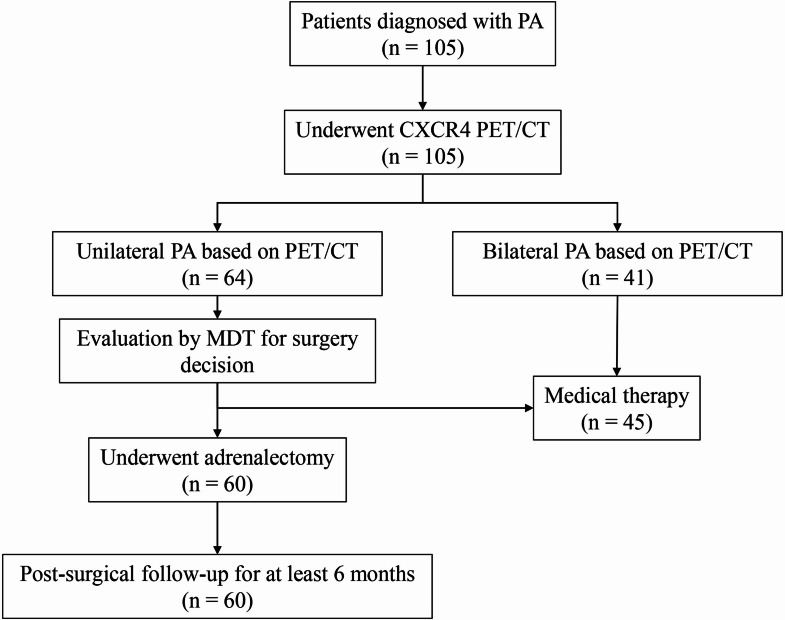
Flowchart of enrolled patients in study

A total of 105 patients diagnosed with PA were enrolled as shown in Fig. 1. Among these patients, 60 who underwent adrenalectomy with at least 6 months post-surgical follow-up were included for analysis. 21 of them underwent AVS successfully. 14 showed significantly unilateral with post ACTH stimulation LI ≥ 4, and 12 appeared contralateral suppression. After adrenalectomy, 50 patients were pathologically diagnosed with APA, while 10 with multifocal aldosterone-producing nodule and/or micronodule (MAPN/ MAPM) according to the histology of primary aldosteronism (HISTALDO) consensus [18, 19]. The median of SUVmax, LLR, and LCR values in APA patients were 9.6, 6.4, and 3.9, respectively. In comparison, the SUVmax, LLR, and LCR values in MAPN/MAPM patients were 9.4, 7.4, and 2.1, respectively. Detailed baseline characteristics are shown in Tables 1 and 2.

**Table 1:** Clinical characteristics and PET/CT values of enrolled patients

Patient Features	Overall Cohort (*n* = 60)	High LCR (*n* = 30)	Low LCR (*n* = 30)	*P* value
Gender				0.0014
Male	35 (58.3%)	11 (36.7%)	24 (80%)	
Female	25 (41.7%)	19 (63.3%)	6 (20%)	
Age (years)	51.0 (40.0-58.5)	51 (41.8–59.0)	50.5 (37.3–56.8)	0.5154
BMI (kg/m^2^)	23.2 (21.3–25.9)	22.9 (20.9–26.0)	23.75 (21.7–25.8)	0.2432
Duration of Hypertension (year)	10.0 (4.3–15.8)	8.0 (4.0-14.3)	12.5 (5.3–18.0)	0.2335
Duration of Hypokalemia (year)	0.5 (0.02-2.0)	0.3 (0.0-3.3)	0.5 (0.1-2.0)	0.4756
Serum Potassium (mmol/L)	3.1 (2.8–3.4)	3.1 (2.8–3.6)	3.0 (2.8–3.3)	0.3334
ARR ([ng/dL]/[ng/mL/h])	304.3 (119.1-783.5)	397.0 (131.8–1067.0)	177.5 (115.2-466.6)	0.0878
PAC (ng/dL)	28.8 (21.4–41.5)	33.1 (22.8–51.5)	26.2 (18.0-35.1)	0.0201
Systolic Pressure (mmHg)	164.0 (156.8-179.5)	160.0 (150.0-176.5)	169.5 (160.0-180.0)	0.0575
Diastolic Pressure (mmHg)	110.0 (98.3–120.0)	103.5 (97.8–115.0)	110.0 (99.0-121.0)	0.2315
AVS (*n* = 21)				
LI	7.5 (3.8–17.7)	21.3 (5.8–41.4)	6.1 (2.8–13.6)	0.0309
CSI	0.9 (0.7-2.0)	0.5 (0.2–1.2)	1.0 (0.8–2.2)	0.0102
Pathological Subtype				0.0122
APA	50 (83.3%)	29 (96.7%)	21 (70.0%)	
MAPN/MAPM	10 (16.7%)	1 (3.3%)	9 (30.0%)	
SUVmax	9.6 (7.1–14.6)	12.1 (9.3–18.5)	8.0 (5.0–12.0)	0.0006
LLR	6.4 (4.6–8.9)	8.0 (4.8–9.8)	6.1 (4.4–7.5)	0.0928
LCR	3.3 (2.0-5.2)	5.2 (4.3–8.1)	2.0 (1.7–2.7)	< 0.0001

IQR = interquartile range. Numbers represent median (IQR) or N (%)

**Table 2:** Clinical characteristics of enrolled patients between different pathological subtypes

Patient Features	APA (*n* = 50)	MAPN/MAPM (*n* = 10)	*P* value
Gender			0.0354
Male	26 (52.0%)	9 (90.0%)	
Female	24 (48.0%)	1 (10.0%)	
Age (years)	51.5 (40.0–59.0)	50.0 (31.8–56.0)	0.5407
BMI (kg/m^2^)	23.12 (21.0-25.4)	22.9 (22.0-29.2)	0.4901
Duration of Hypertension (year)	10.0 (3.8–15.3)	12.0 (5.5–18.5)	0.5273
Duration of Hypokalemia (year)	0.5 (0.0-2.3)	1.5 (0.4–2.3)	0.2881
Serum Potassium (mmol/L)	3.0 (2.8–3.4)	3.1 (2.7–3.4)	0.965
ARR ([ng/dL]/[ng/mL/h])	294.1 (117.2-800.5)	361.3 (152.2–1326.0)	0.6744
PAC (ng/dL)	29.8 (21.6–42.8)	21.7 (15.8–35.2)	0.1319
Systolic Pressure (mmHg)	163.5 (153.8–180.0)	165.0 (160.0-170.0)	0.918
Diastolic Pressure (mmHg)	110.0 (96.8–120.0)	108.0 (99.5–120.0)	0.8635
AVS (*n* = 21)			
LI	12.7 (3.8–20.2)	6.2 (2.9–7.3)	0.3168
CSI	0.8 (0.6–1.6)	1.8 (1.0-2.7)	0.0304
SUVmax	9.6 (7.2–16.2)	9.4 (6.0-12.5)	0.6147
LLR	6.4 (4.4–8.9)	7.4 (5.4–9.8)	0.3702
LCR	3.9 (2.3-6.0)	2.1 (1.8–2.8)	0.0055

IQR = interquartile range. Numbers represent median (IQR) or N (%)

### LCR holds predictive value for clinical outcome in patients with PA after adrenalectomy

At the 3-month mark, 31 patients achieved complete clinical success, and 29 achieved partial clinical success. Similarly, at the 6-month assessment, 35 patients achieved complete clinical success, while 25 achieved partial clinical success.

At the 3-month mark, following univariate logistic screening, the duration of hypertension (OR = 0. 906, 95% CI 0.837–0.980; *p* = 0.014) and LCR (OR = 2.785, 95% CI 1.628–4.766; *p* = 0.0002) were incorporated into the multivariate logistic regression model (Supplementary Table 1). Subsequently, LCR (OR = 2.900, 95% CI 1.625–5.177; *p* = 0.0003) and the duration of hypertension (OR = 0.908, 95% CI 0.823–1.002; *p* = 0.055) emerged as independent predictors of clinical success at the 3-month assessment (Supplementary Table 2). At the 6-month interval, after univariate logistic screening, BMI (OR = 0.853, 95% CI 0.729–0.998; *p* = 0.047), duration of hypertension (OR = 0.890, 95% CI 0.819–0.966; *p* = 0.005), and LCR (OR = 2.227, 95% CI 1.400-3.542; *p* = 0.0007) were included in the multivariate logistic regression model (Supplementary Table 3). Consequently, LCR (OR = 2.232, 95% CI 1.345–3.702; *p* = 0.002) and the duration of hypertension (OR = 0.898, 95% CI 0.815–0.989; *p* = 0.028) were identified as independent predictors of clinical success at the 6-month assessment (Supplementary Table 4).

**Fig. 2:**
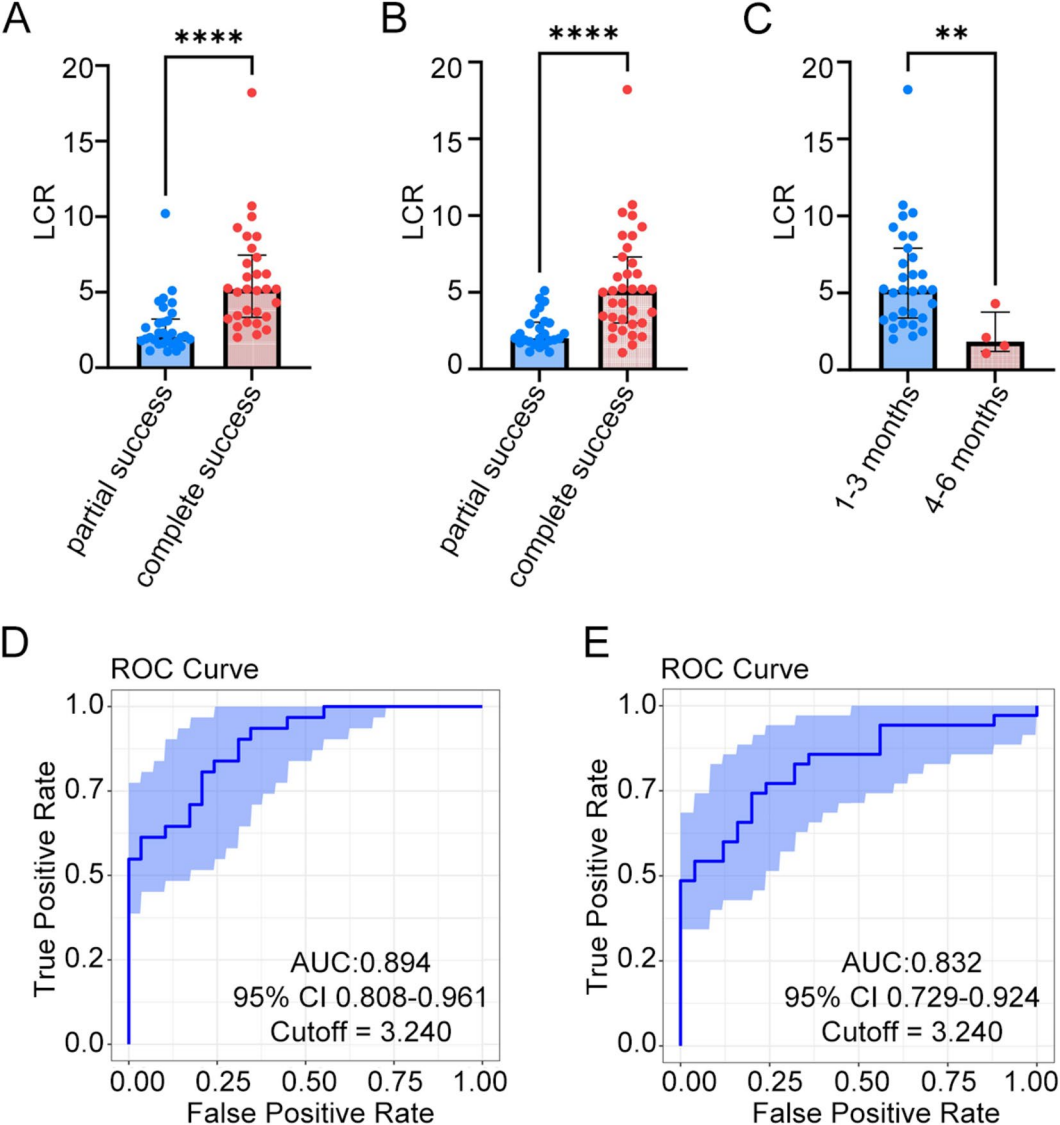
The accuracy of LCR in predicting clinical outcomes. **(A-B)** Comparison of LCR between partial and complete success patients at 3 months **(A)** and 6 months **(B)** assessment by Mann-Whitney test. **(C)** Comparison of LCR between patients who achieved complete clinical success 1–3 months and 4–6 months after adrenalectomy by Mann-Whitney test. **(D)** ROC curve of LCR to predict 3-month clinical outcome, with AUC of 0.894 (95% CI 0.808–0.961) and cut-off of 3.240. **(E)** ROC curve of LCR to predict 6-month clinical outcome, with AUC of 0.832 (95% CI 0.729–0.924) and cut-off of 3.240. ** *p* < 0.01, **** *p* < 0.0001

We also observed that the LCR was higher in patients who achieved complete clinical success compared to those with partial success at both the 3-month (Fig. 2A) and 6-month assessments (Fig. 2B). Additionally, among patients who achieved complete clinical success, those achieving it within 1–3 months exhibited significantly higher LCR than those achieving it within 4–6 months (Fig. 2C).

**Fig. 3:**
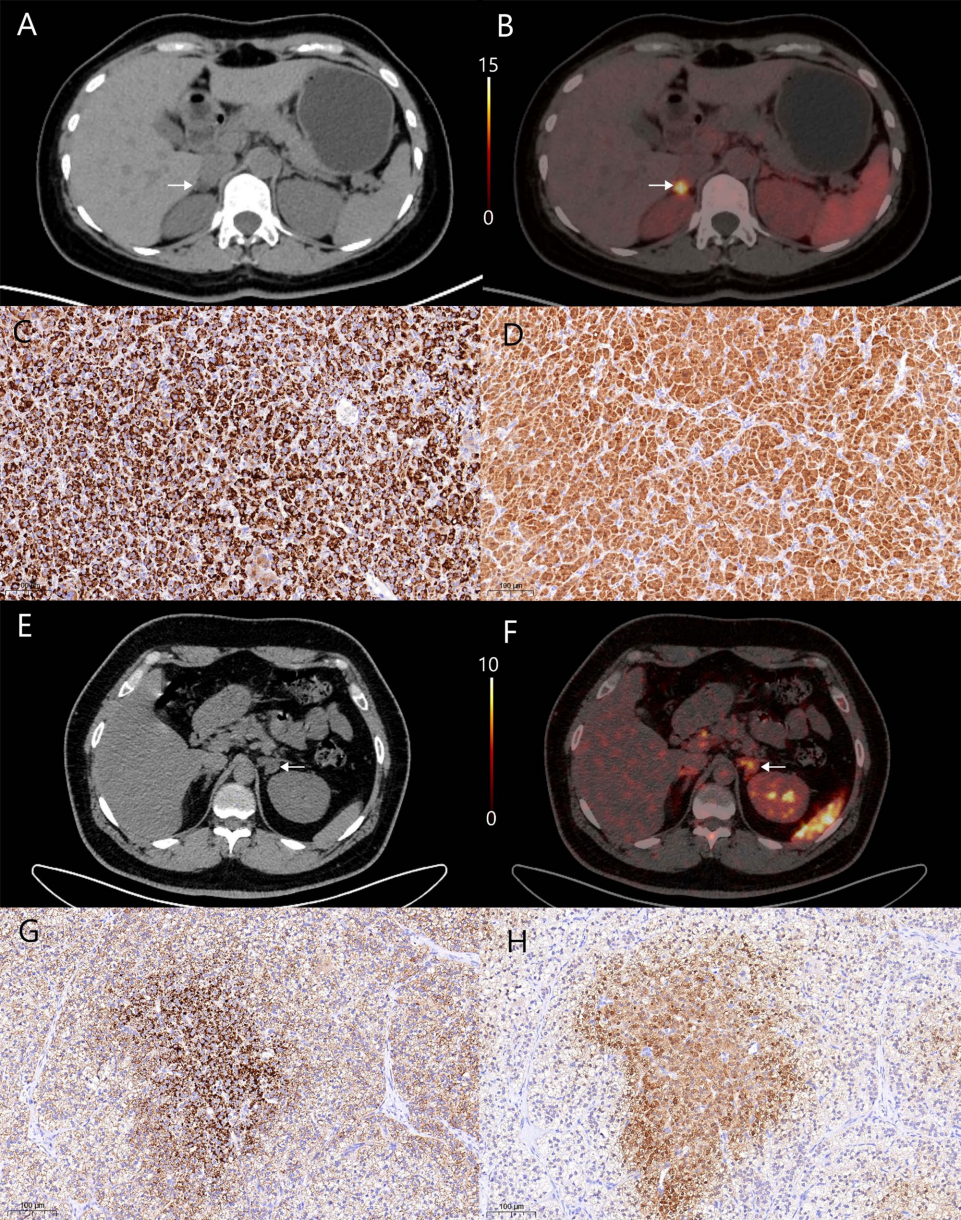
Imaging performance of CXCR4 PET/CT and immunohistochemistry in UPA patients. **A-D** A 60-year-old female of high LCR group, CT **(A)** and PET/CT **(B)** indicated UPA on the right side (SUVmax of 14.42, LLR of 8.85, LCR of 6.19). The expression level of both CXCR4 **(C)** and CYP11B2 **(D)** was shown by immunohistochemistry. **(E-H)** A 35-year-old male of low LCR group, CT **(E)** and PET/CT **(F)** indicated UPA on the left side (SUVmax of 8.49, LLR of 6.48, LCR of 1.89). Immunohistochemistry showed the expression level of both CXCR4 **(G)** and CYP11B2 **(H).** Lesions were indicated by white arrows in CT and PET/CT imaging. Magnification x20 in immunohistochemical staining

Then we employed ROC analysis to assess the accuracy of LCR in predicting clinical success and identify the optimal cut-off value. And 1000 bootstrap samples were used for internal validation. At the 3-month mark, the AUC for LCR is 0.894 (95% CI 0.808–0.961), with an optimal cut-off of 3.240 (Fig. 2D). By the 6-month assessment, the AUC for LCR had decreased to 0.832 (95% CI 0.729–0.924), while the optimal cut-off remained at 3.240 (Fig. 2E). Patients with LCR > 3.240 were classified into the high LCR group (*n* = 30), while those with LCR ≤ 3.240 comprised the low LCR group (*n* = 30). The imaging performance of CXCR4 PET/CT combined with immunohistochemistry in representative patients of high and low LCR groups is shown in Fig. 3.

### High LCR group demonstrates superior clinical outcome compared to low LCR group

**Fig. 4:**
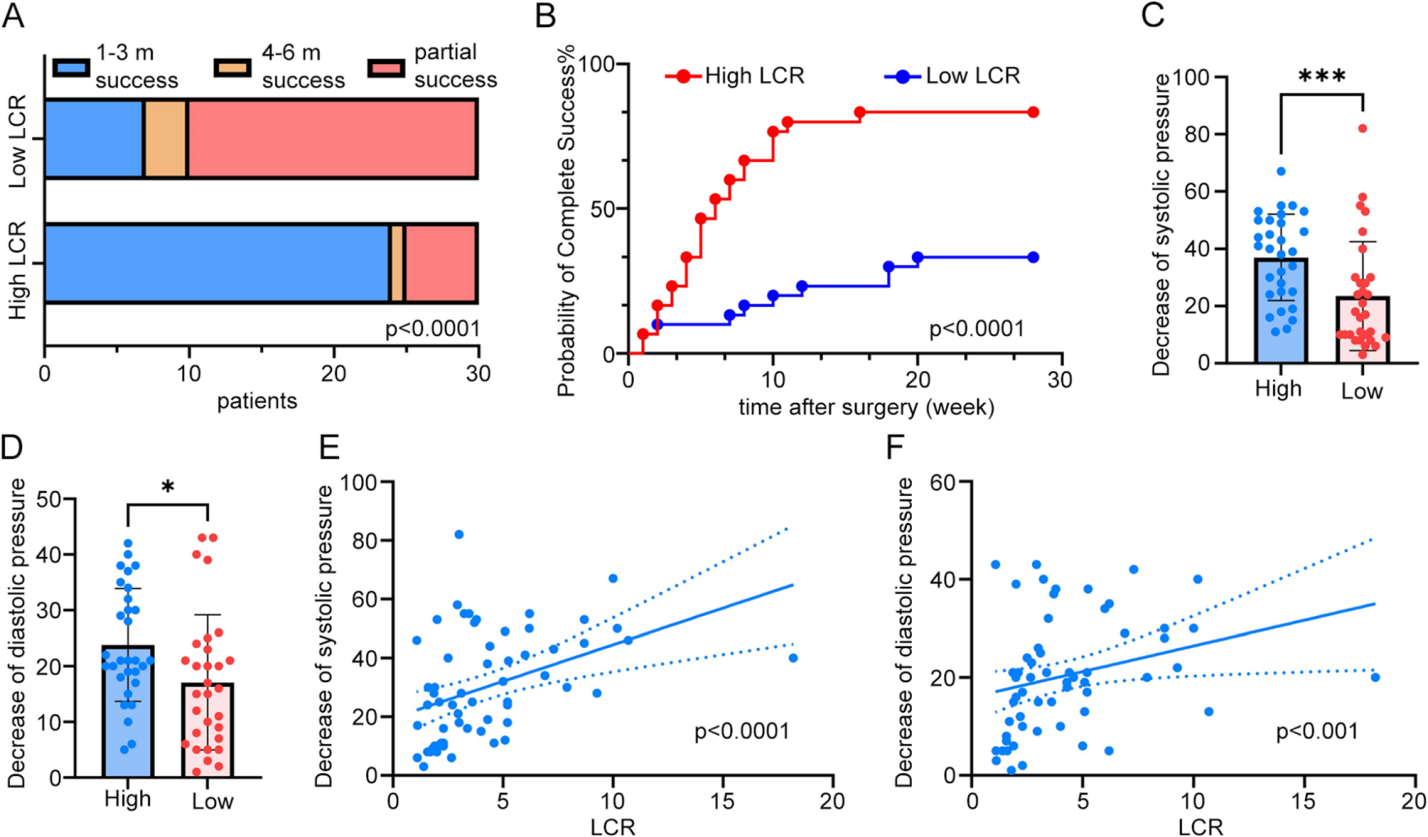
High LCR group demonstrates superior clinical outcome compared to low LCR group. **(A)** The high LCR group exhibited a significantly greater number of patients achieving complete clinical success compared to the low LCR group (Fisher exact test, *P* < 0.0001). Blue represents patients achieving complete clinical success 1–3 months after surgery, brown represents patients achieving complete clinical success 4–6 months after surgery, while red represents patients of partial clinical success 6 months after surgery. **(B)** Kaplan–Meier analysis of the probability of attaining complete clinical success between the high and low LCR groups. **(C-D)** Comparison of systolic pressure **(C)** and diastolic pressure **(D)** decrease between high and low LCR groups by Mann-Whitney test. **(E-F)** Correlation analysis between LCR and decrease of systolic pressure **(E)** and diastolic pressure **(F)** by Spearman. * *p* < 0.05, *** *p* < 0.001

The high LCR group had a 3-month complete clinical success rate of 80.00%, while the low LCR group had a rate of 23.33%. At 6 months, the high LCR group had a complete clinical success rate of 83.33%, while the low LCR group had a rate of 33.33%. The high LCR group had significantly more patients achieving complete clinical success compared to the low LCR group (Fig. 4A). Additionally, the high LCR group had a significantly higher probability of achieving complete clinical success compared to the low LCR group (Fig. 4B). We also compared the blood pressure reduction 6 months after surgery between the high LCR and low LCR groups, revealing a significantly greater reduction of systolic pressure (Fig. 4C) and diastolic pressure (Fig. 4D) in the high LCR group. Moreover, a significant positive correlation by Spearman between LCR and systolic pressure (Fig. 4E) and diastolic pressure (Fig. 4F) reduction was discovered.

### High LCR indicates greater aldosterone-producing function of the lesion

**Fig. 5:**
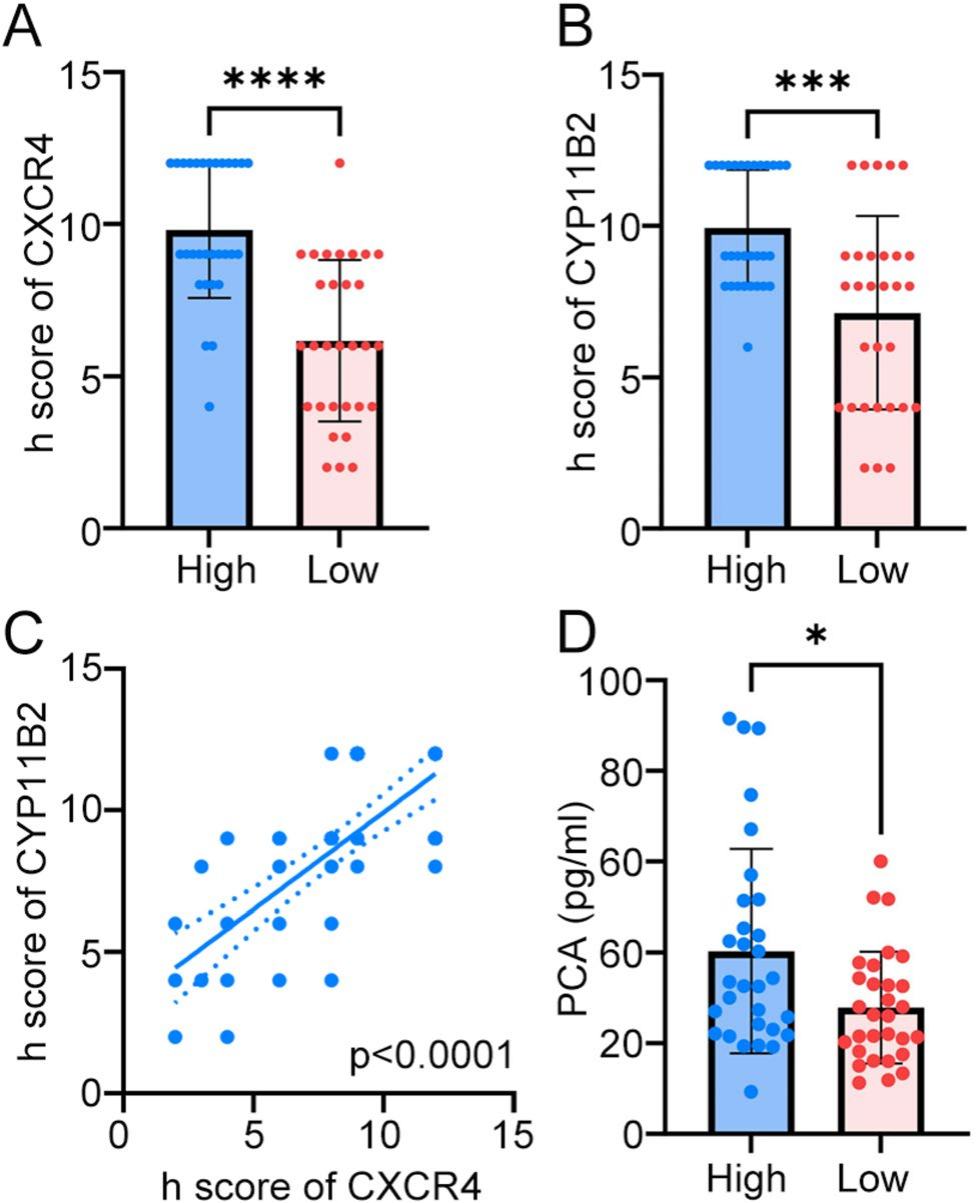
High LCR indicates greater aldosterone-producing function. **(A-B)** Comparison of CXCR4 **(A)** and CYP11B2 **(B)** expression with h score between high and low LCR groups by Mann-Whitney test. **(C)** Correlation analysis between expression level of CXCR4 and CYP11B2 by Spearman. **(D)** Comparison of preoperative plasma aldosterone concentration (PAC) level between high and low LCR groups by Mann-Whitney test. * *p* < 0.05, *** *p* < 0.001, **** *p* < 0.0001

Immunohistochemical analysis for CXCR4 and CYP11B2 was performed in postoperative adrenal tissue sections from all patients. Compared with the low LCR group by a semi-quantitative h score, the high LCR group exhibited elevated CXCR4 (Fig. 5A) and CYP11B2 expression (Fig. 5B). Moreover, the expression level of CXCR4 and CYP11B2 are positively correlated (Fig. 5C). Results of correlation analyses between LCR and clinical features were shown in Table 3. A significant positive correlation between LCR and PAC was observed. Also, the high LCR group showed higher PAC level than the low LCR group (Fig. 5D).

**Table 3:** Correlation analyses of LCR with clinical features among enrolled patients

Patient Features	Spearman’s ρ	*P* value
Age (years)	0.092	0.484
BMI (kg/m^2^)	-0.192	0.142
Duration of Hypertension (year)	-0.189	0.148
Duration of Hypokalemia (year)	-0.110	0.404
Serum Potassium (mmol/L)	0.067	0.612
ARR (pg/ml/uIU/mL)	0.133	0.312
PAC (pg/mL)	0.277	0.033
Systolic Pressure (mmHg)	-0.083	0.529
Diastolic Pressure (mmHg)	-0.019	0.886
LI	0.604	0.004
CSI	-0.656	0.001

### LCR suggests level of lateralization and contralateral suppression

**Fig. 6:**
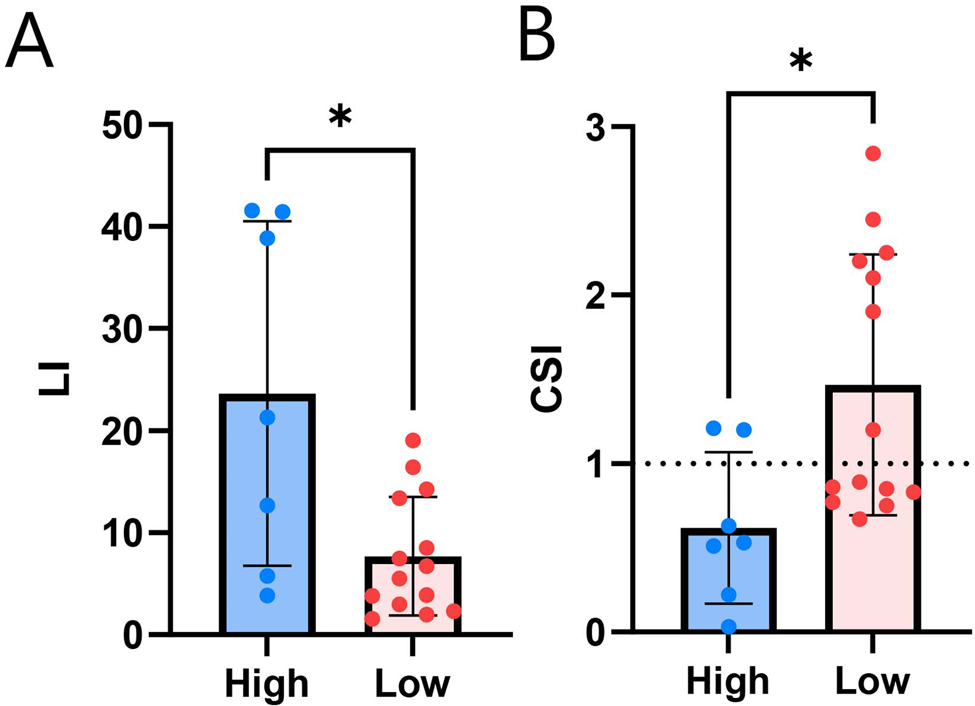
LCR suggests level of lateralization and contralateral suppression. **(A-B)** Comparison of LI **(A)** and CSI **(B)** between high and low LCR groups by Mann-Whitney test. * *p* < 0.05

The relationship between LCR and AVS parameters was analyzed. Our findings indicated that LCR is positively correlated with LI and negatively correlated with CSI, as presented in Table 3. Furthermore, the high LCR group exhibited significantly higher LI (Fig. 6A) and lower CSI (Fig. 6B).

## Discussion

As a safe, non-invasive test with high accuracy, CXCR4 PET/CT requires no special preparation and has been demonstrated with stable capability in PA diagnosis and subtyping [11, 12]. Despite extensive research on the use of CXCR4 PET/CT in the clinical management of PA [20], the prognostic potential of uptake values has not been fully evaluated. Although previous studies suggested that high CXCR4 PET/CT uptake values correlate with better postoperative outcomes, they failed to conclusively confirm the predictive value and accuracy of these values for clinical outcomes [12–14, 21]. In our previous study, we compared the subtyping accuracy of PA between CXCR4 PET/CT and AVS. But due to the limitation of patient number, how much can CXCR4 PET/CT predict the biochemical and clinical outcomes was not fully explored [15]. To further explore the prognostic value of CXCR4 PET/CT, we conducted a prospective cohort study over a longer period with a larger patient cohort. In this study, we demonstrated the predictive value of CXCR4 PET/CT for postoperative blood pressure recovery in patients with UPA. Additionally, we established a cut-off value to identify UPA patients who are most likely to benefit from adrenalectomy.

After performing univariate and multivariate logistic regression analyses, we found that clinical outcome for unilateral primary aldosteronism patients after adrenalectomy was significantly associated with the duration of hypertension and the LCR at 3 and 6 months. Several studies have reported that patients aged 50 years or younger with hypertension lasting less than 5 years before adrenalectomy are more likely to achieve normal blood pressure postoperatively [22, 23]. It has been suggested that long-standing hypertension, despite surgical correction of hormonal abnormalities, may cause irreversible vascular damage. This irreversible damage likely explains why duration of hypertension is considered a risk factor for poorer clinical outcomes [22].

As depicted above, LCR serves as an independent predictor for both the 3-month and 6-month clinical outcomes in multivariate logistic regression. Patients with a high LCR are more likely to achieve complete clinical success. Additionally, the 3-month (AUC = 0.929) and 6-month (AUC = 0.806) ROC curves also demonstrated the satisfactory accuracy of LCR in predicting clinical outcomes. For clinical guidance, we determined the LCR cut-off (3.240) and categorized patients into low and high LCR groups based on this threshold. Patients with high LCR according to the cutoff showed greater probability to achieve complete clinical success and larger blood pressure reduction.

Curiously, although LCR was recognized as an independent predictor of clinical outcomes, neither SUVmax nor LLR demonstrated predictive ability for clinical success. This could be attributed to the fact that both SUVmax and LLR only indicate the ^68^Ga-Pentixafor uptake level of the lesion, without comparing uptake values between the lesion and the contralateral adrenal gland. In contrast, LCR represents the ratio of lesion SUVmax to contralateral adrenal tissue SUVmean, incorporating both the heightened expression level in the lesion and the baseline in the contralateral adrenal of CXCR4. Given the strong correlation between CXCR4 and aldosterone synthase CYP11B2 [10], LCR provides a more accurate representation of the lesion’s functional superiority over the contralateral adrenal gland. Therefore, LCR is expected to be a more predictive indicator of the clinical outcome than SUVmax and LLR, a notion strongly supported by our results. Additionally, in a previous study, the lateralization index based on SUVmax (ratio of dominant side SUVmax to nondominant side SUVmax) performed better in subtyping of PA compared to SUVmax and LLR [11]. This finding underscores the importance of accounting for lateralization in the analysis of CXCR4 PET/CT imaging in PA.

In this study, we also sought to establish a theoretical foundation for using LCR as a predictor of postoperative clinical outcomes in UPA. Our results revealed that the high LCR group exhibited elevated h scores for CXCR4 and CYP11B2, along with a higher PAC level compared to the low LCR group. Additionally, we found a significant positive correlation between LCR and PAC. Considering adrenal physiology, the unrestricted expression of CYP11B2 and aldosterone secretion inhibits the renin-angiotensin-aldosterone system (RAAS). The low angiotensin II level subsequently reduces CYP11B2 expression in the normal tissue of the contralateral adrenal gland [24]. Consequently, the normal tissue of the contralateral adrenal exhibits less uptake in CXCR4 PET/CT. A low SUVmean in the contralateral adrenal indicates controlled function and contralateral suppression, as evidenced by elevated LCR. Furthermore, we found that LCR is positively correlated with LI and negatively correlated with CSI. The prognostic value of contralateral suppression has been shown in previous studies, indicating that its presence may suggest a better outcome [25–28]. Taken together, LCR represents the functional level of aldosterone production and indicates the degree of lateralization and contralateral suppression. Therefore, it is not surprising that LCR serves as an independent predictor of clinical outcomes after surgery.

Our study also demonstrated that APA patients had significantly higher LCR than MAPN/MAPM patients (Table 2), indicating the potential value of CXCR4 PET/CT in pathological subtype diagnosis of primary aldosteronism. But the mechanism of this different uptake remains unclear, and further investigation is needed.

The KCNJ5 gene mutation is the most frequently observed somatic mutation in APA patients. This mutation has been shown to activate calcium signaling pathways, resulting in increased expression of CYP11B2 and subsequent aldosterone overproduction [29]. Patients harboring the KCNJ5 mutation exhibit significantly higher expression levels of aldosterone synthase (CYP11B2) compared to those with the wild-type KCNJ5 gene [30]. Previous studies have demonstrated that patients with KCNJ5 mutations exhibit a higher PET-positive rate and greater uptake of ^68^Ga-pentixafor compared to patients with wild-type KCNJ5. Furthermore, the rates of clinical and biochemical complete remission were significantly higher in patients harboring the KCNJ5 mutation than in those with the wild-type KCNJ5 gene [31, 32].

High CXCR4 expression has also been demonstrated in cortisol-producing adenomas [33]. Although the exact mechanism remains unclear, previous studies have indicated that abnormal expression and function of G protein-coupled receptors (GPCRs) in adrenal glands of patients with adrenal adenomas or adrenocorticotropic hormone-independent macronodular adrenal hyperplasia can lead to dysregulated cell proliferation and steroid synthesis [34]. CXCR4 is a typical member of the GPCR family, which may explain why ^68^Ga-pentixafor uptake occurs in cortisol-producing adenomas.

**Fig. 7:**
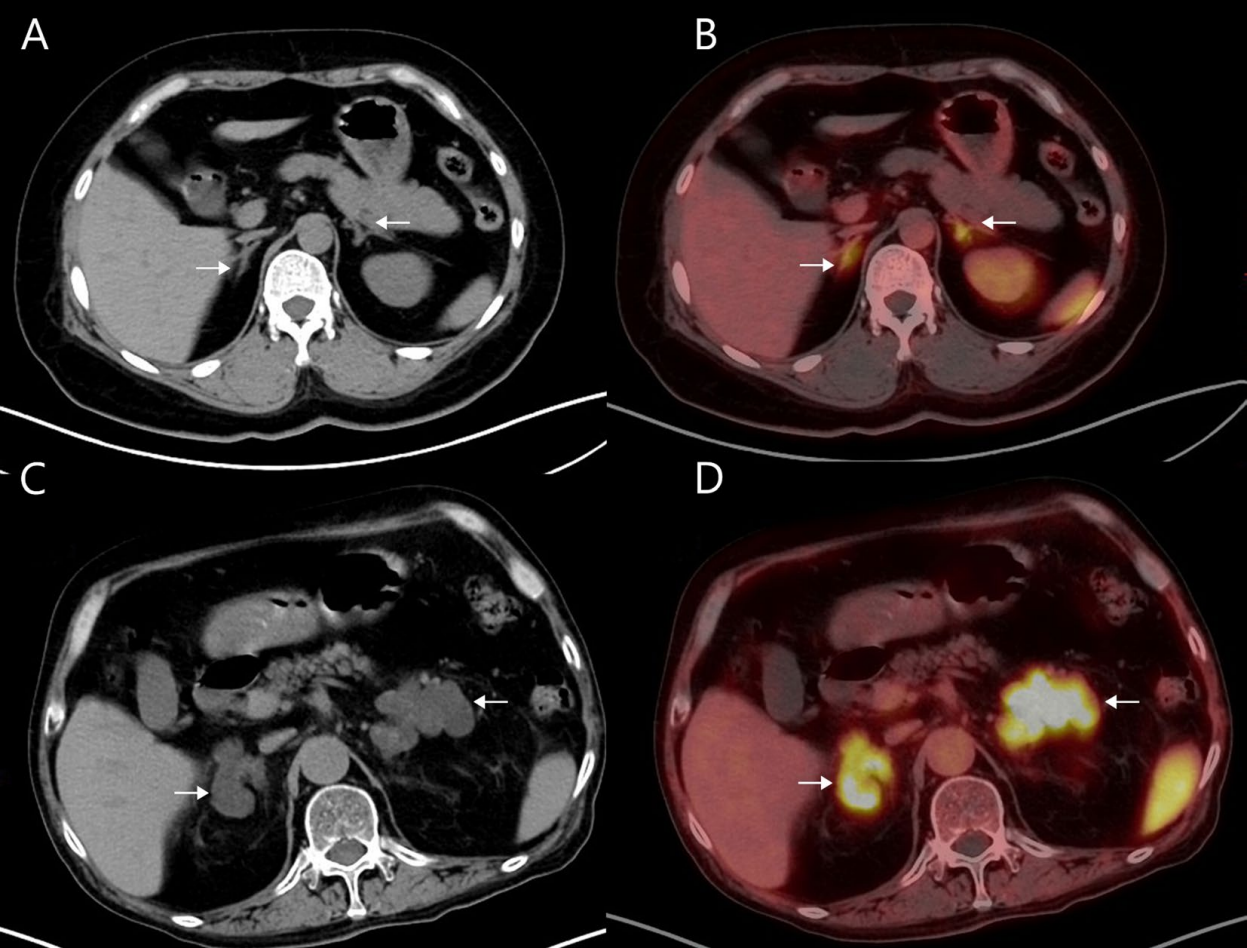
CXCR4-PET/CT images from one patient with Cushing’s disease caused by a pituitary tumor and one patient with adrenal macronodular hyperplasia. **(A-B)** Cushing’s disease (pituitary tumor). The patient exhibited no Cushingoid facies, with a BP of 118/75 mmHg, cortisol level of 530.8 nmol/L, and ACTH level of 72.9 ng/L. PET imaging revealed mild, uneven thickening of both adrenal glands, with a maximum diameter of 7 mm. On the left side, SUVmax was 4.9, and SUVavg was 3.1. On the right side, SUVmax was 4.8, and SUVavg was 2.9, with no significant asymmetry observed. **(C-D)** Cushing’s syndrome. The patient presented with central obesity, a full moon face, and a BP of 172/102 mmHg. Cortisol levels were 1468.2 nmol/L, and ACTH levels were 6.3 ng/L. The left adrenal gland exhibited a large lesion, measuring 56 × 40 mm, with an SUVmax of 13.4 and an SUVavg of 8.5. The right adrenal lesion measured 39 × 38 mm, with an SUVmax of 10.3 and an SUVavg of 6.6. The left adrenal gland was more dominant, leading to a subsequent left adrenalectomy. Following surgery, BP decreased but did not return to normal

We attempted to identify patients with elevated cortisol levels who underwent CXCR4-PET/CT examinations at participating institutions over the past 5 years. However, we did not find any cases of cortisol-producing adenomas; instead, we identified one patient with Cushing’s disease caused by a pituitary tumor and one patient with adrenal macronodular hyperplasia. Figure 7 illustrated representative CXCR4-PET/CT images from these patients. Given the limited number of cases, we could not determine whether a significant difference exists in ^68^Ga-pentixafor uptake between patients with primary and secondary hypercortisolism.

It is noteworthy that our follow-up assessment incorporated biochemical outcomes. However, we found that out of the 60 patients, only 3 did not attain complete success, with the remaining 57 achieving complete success. This rendered statistical analysis irrelevant; therefore, we did not investigate the predictive capability of CXCR4 PET/CT for biochemical outcomes in this study.

There are some limitations of this study: (1) This is a single-center prospective cohort with a small sample size and lack of external validation. And due to the limited patient number, we could not analyze the predictive value of CXCR4 PET/CT for biochemical outcome. (2) The follow-up was not long enough. A meta-analysis showed that the proportion of PA patients who achieved complete clinical success dropped 6.7% per year [35]. Due to the limited follow-up period, we could not evaluate the value of CXCR4 PET/CT in predicting hypertension recurrence after adrenalectomy. A larger cohort with years of follow-up should be performed focusing on the prognostic value of CXCR4 PET/CT.

Despite these limitations, this is the first study to prove that LCR of CXCR4 PET/CT is an independent predictive factor for clinical outcomes. Moreover, a cut-off was determined for clinical guidance. The preliminary theoretical foundation was provided for the clinical usage of CXCR4 PET/CT in PA patients.

## Conclusions

LCR is a reliable independent predictor of postoperative blood pressure recovery in PA. Patients with LCR over 3.240 may benefit more from adrenalectomy. We recommend increased utilization of CXCR4 PET/CT for patients with PA. This non-invasive test not only offers a stable typing ability for PA, but also can accurately predict postoperative blood pressure recovery in patients with UPA.

## Electronic supplementary material

Below is the link to the electronic supplementary material.

Supplementary Material 1

## Data Availability

The datasets generated or analyzed during the study are available from the corresponding author on reasonable request.

## Abbreviations

PA: Primary aldosteronism
UPA: Unilateral primary aldosteronism
APA: Aldosterone-producing adenomas
ARR: Aldosterone-to-renin ratio
PAC: Plasma aldosterone concentration
AVS: Adrenal vein sampling
PASO: Primary Aldosteronism Surgical Outcome
LI: Lateralization index
CSI: Contralateral suppression index
SUVmax: Maximum standardized uptake values
LLR: The lesional SUVmax to the normal liver SUVmean ratio
LCR: The lesional SUVmax to the contralateral adrenal tissue SUVmean ratio
