# Supplementary material for: The prognostic value of CXCR4 PET/CT imaging in unilateral primary aldosteronism patients after adrenalectomy
Source: EJNMMI Res. 2025 Apr 17;15:41. doi: 10.1186/s13550-025-01242-6 (PMC12006604; doi:10.1186/s13550-025-01242-6)
Supplement: Supplementary file 1 — Supplementary Material 1 [file 13550_2025_1242_MOESM1_ESM.docx]

**Supplementary Table 1 Univariate logistic regression of 3-month clinical outcome.**

| **Variable** | **OR** | **95%CI** | **p value** |  |
| --- | --- | --- | --- | --- |
| Female Sex | 1.348 | (0.481-3.779) | 0.571 |  |
| Age, y | 0.994 | (0.950-1.041) | 0.807 |  |
| BMI | 0.903 | (0.778-1.050) | 0.185 |  |
| Duration of Hypertension, y | 0.906 | (0.837-0.980) | 0.014 | * |
| Duration Hypokalemia, y | 1.090 | (0.925-1.283) | 0.304 |  |
| Serum Potassium | 0.778 | (0.317-1.905) | 0.582 |  |
| Aldosterone-to-Renin Ratio | 1.000 | (1.000-1.001) | 0.432 |  |
| Plasma Aldosterone Concentration | 1.024 | (0.993-1.055) | 0.129 |  |
| Systolic Pressure | 1.006 | (0.972-1.041) | 0.730 |  |
| Diastolic Pressure | 1.017 | (0.978-1.057) | 0.391 |  |
| LLR | 1.162 | (0.984-1.372) | 0.076 |  |
| SUVmax | 1.072 | (0.994-1.156) | 0.073 |  |
| LCR | 2.785 | (1.628-4.766) | 0.0002 | *** |

** *p* < 0.01, *** *p* < 0.001

**Supplementary Table 2 Multivariate logistic regression of 3-month clinical outcome.**

| **Variable** | **OR** | **95%CI** | **p value** |  |
| --- | --- | --- | --- | --- |
| Duration of Hypertension, y | 0.908 | (0.823-1.002) | 0.055 |  |
| LCR | 2.900 | (1.625-5.177) | 0.0003 | *** |

*** *p* < 0.001

**Supplementary Table 3 Univariate logistic regression of 6-month clinical outcome.**

| **Variable** | **OR** | **95%CI** | **p value** |  |
| --- | --- | --- | --- | --- |
| Female Sex | 1.497 | (0.522-4.292) | 0.453 |  |
| Age, y | 0.991 | (0.946-1.038) | 0.712 |  |
| BMI | 0.853 | (0.729-0.998) | 0.047 | * |
| Duration of Hypertension, y | 0.890 | (0.819-0.966) | 0.005 | ** |
| Duration Hypokalemia, y | 1.055 | (0.897-1.241) | 0.519 |  |
| Serum Potassium | 0.632 | (0.243-1.643) | 0.346 |  |
| Aldosterone-to-Renin Ratio | 1.000 | (1.000-1.001) | 0.285 |  |
| Plasma Aldosterone Concentration | 1.025 | (0.993-1.058) | 0.130 |  |
| Systolic Pressure | 1.008 | (0.974-1.044) | 0.636 |  |
| Diastolic Pressure | 1.018 | (0.979-1.059) | 0.379 |  |
| LLR | 0 | (0.905-1.502) | 0.235 |  |
| SUVmax | 1.045 | (0.979-1.115) | 0.189 |  |
| LCR | 2.227 | (1.400-3.542) | 0.0007 | *** |

* *p* < 0.05, ** *p* < 0.01, *** *p* < 0.001

**Supplementary Table 4 Multivariate logistic regression of clinical outcome 6 month after adrenalectomy.**

| **Variable** | **OR** | **95%CI** | **p value** |  |
| --- | --- | --- | --- | --- |
| BMI | 0.908 | (0.753-1.094) | 0.303 |  |
| Duration of Hypertension, y | 0.898 | (0.815-0.989) | 0.028 | * |
| LCR | 2.232 | (1.345-3.702) | 0.002 | ** |

* *p* < 0.05, ** *p* < 0.01
